# Supplementary material for: Venomix: a simple bioinformatic pipeline for identifying and characterizing toxin gene candidates from transcriptomic data
Source: PeerJ. 2018 Jul 31;6:e5361. doi: 10.7717/peerj.5361 (PMC6074769; doi:10.7717/peerj.5361)
Supplement: Supplemental Information 3 [file peerj-06-5361-s003.gz › FinalOutput_E-6/Cysteine-rich_venom_protein_asurin-1_1/finaltree.pdf]

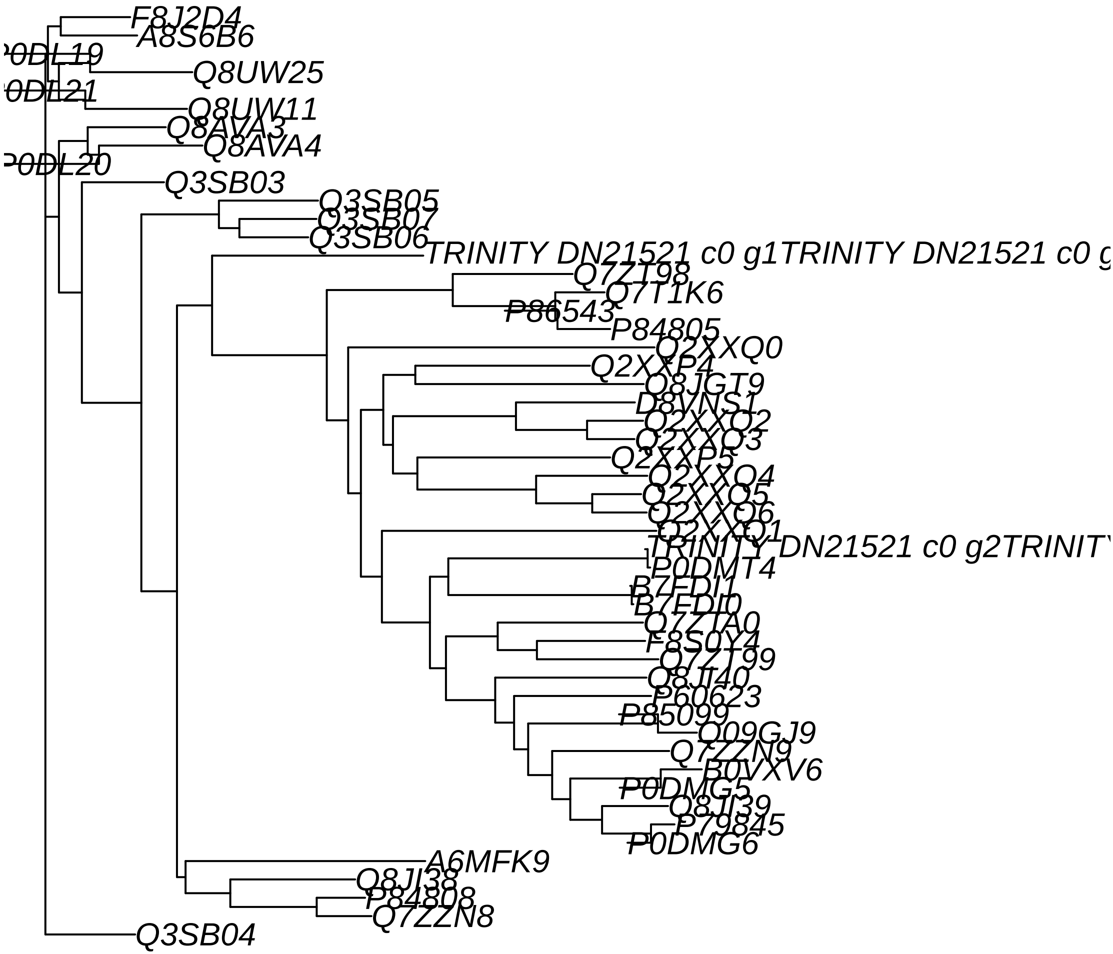
F8J2D4
A8S6B6
P0DL19
Q8UW25
P0DL21
Q8UW11
Q8AVA3
Q8AVA4
P0DL20
Q3SB03
Q3SB05
Q3SB07
Q3SB06
TRINITY DN21521 c0 g1TRINITY DN21521 c0 g
Q7Z198
Q7T1K6
P86543
P84805
Q2XXQ0
Q2XXP4
Q8JGT9
D8VNS1
Q2XXQ2
Q2XXQ3
Q2XXP5
Q2XXQ4
Q2XXQ5
Q2XXQ6
Q2XXQ1
TRINITY DN21521 c0 g2TRINITY
P0DMT4
B7FDI1
B7FDI0
Q7ZTA0
F8S0Y4
Q7ZT99
Q8JI40
P60623
P85099
Q09GJ9
Q7ZZN9
B0VXV6
P0DMG5
Q8JI39
P79845
P0DMG6
A6MFK9
Q8JI38
P84808
Q7ZZN8
Q3SB04
